# Gastrointestinal helminth parasites of pets: Retrospective study at the veterinary teaching hospital, IPB University, Bogor, Indonesia

**DOI:** 10.14202/vetworld.2023.1043-1051

**Published:** 2023-05-17

**Authors:** Yusuf Ridwan, Etih Sudarnika, Tri Isyani Tungga Dewi, Novericko Ginger Budiono

**Affiliations:** 1 Division of Parasitology and Medical Entomology, School of Veterinary Medicine and Biomedical Sciences, IPB University, Bogor, Indonesia; 2 Division of Veterinary Public Health and Epidemiology, School of Veterinary Medicine and Biomedical Sciences, IPB University, Bogor, Indonesia; 3 Veterinary Teaching Hospital, School of Veterinary Medicine and Biomedical Sciences, IPB University, Bogor, Indonesia; 4 Division of Medical Microbiology, School of Veterinary Medicine and Biomedical Sciences, IPB University, Bogor, Indonesia

**Keywords:** Bogor, cat, dog, gastrointestinal helminth, zoonosis

## Abstract

**Background and Aim:**

Dogs and cats are popular pets that play integral roles in human societies worldwide. Unfortunately, they can carry potential zoonotic helminths that can be transmitted to humans. However, data on the gastrointestinal helminths affecting dogs and cats in Bogor, Indonesia, are currently lacking. This study aimed to investigate the occurrence of gastrointestinal helminth parasites in pets from this area using a retrospective analysis.

**Materials and Methods:**

A retrospective study was conducted at the Veterinary Teaching Hospital of IPB University, Bogor. Cat and dog patients from January 2014 to April 2019 were tested for helminth infections and the results as well as their age, sex, and breed data were analyzed using the Chi-square test.

**Results:**

Among the dogs and cats examined for internal parasites, 61.11% (33/51) of the dogs and 53.80% (92/171) of the cats were infected by helminths. Among the dogs, hookworm (37.04%) and Toxocara spp. (24.07%) were detected, while in the cats, hookworm (11.11%), Toxocara spp. (38.01%), and Dipylidium caninum (4.68%) were detected. The prevalence of hookworm and D. caninum was higher in older pets, while Toxocara spp. infected younger cats and dogs (<1 year) more frequently. The prevalence of Toxocara spp. in the Indonesian local dog breed was higher when than other breeds. Sex did not significantly affect the prevalence of parasites in dogs or cats.

**Conclusion:**

The discovery of zoonotic helminth parasites in the cat and dog pets from Bogor raises concerns for the inhabitants. Initiatives will be required to inform pet owners about prevention strategies for these parasitic diseases.

## Introduction

Pet ownership in Indonesia, in cities in particular, has increased over the last decade. A survey has also shown that the rates of cat ownership (37%) have now surpassed dog ownership (16%) [1]. Pet ownership has several acknowledged benefits, such as reducing stress, inducing sympathetic responses, and improving lipid profiles. This is evidenced as cat and dog owners report receiving love, companionship, affection, and company from their pets [2]. Pet owners feel emotionally happy as their pets help them to cope with stress and loneliness [3]. The increase in pet ownership can also be related to using pet animals in cultural services, such as dogs that are used for helping blind people to walk and pets used for pest control [4]. The increase in pet ownership is also related to cultural changes; the development of technical facilities such as pet harnesses and portable cages; and increased online purchasing availability. Furthermore, many shopping centers and outdoor areas in Indonesian cities are now pet friendly. Many pet owners, especially dog and cat owners, reportedly view their pets as family members and companions [5]. Pets also provide companionship when exercising, such as walking, running, playing, or working in a park [6].

Despite the benefits of pet ownership, there are also health hazards to consider, as pets can carry zoonotic diseases that can naturally transmit between vertebrate animals and humans. Zoonotic diseases can be transmitted from animals to humans, and *vice versa*, from humans to animals [7–10]. Animals can carry parasites, including helminths [11–13], that can be transferred to humans through contaminated food [14–16] or contact with contaminated environments [17–19]. Many intestinal parasites in dogs and cats can infect humans if they come into contact with them directly or if people are exposed to areas polluted by their feces [12, 20–30]. Some of the specific parasites that can infect humans include *Toxocara* spp., *Toxascaris* [31, 32], hookworms [33, 34], *Dipylidium caninum* [35–37], and *Strongyloides* spp. [38, 39]. For example, infection from the common roundworms found in dogs and cats, that is, *Toxocara canis* and *Toxocara cati*, can occur through the unintentional consumption of infective eggs and result in visceral and ocular larva migrans syndromes in people [40–42]. The hookworms that infect dogs and cats, mainly *Ancylostoma* spp. can also infect humans with different mechanisms of infection compared to *Toxocara* spp. infections. *Ancylostoma* spp. eggs expelled from cat and dog feces will develop into infective larvae (stadium III) in the environment. Humans can get the infection by hookworms when the infective larvae of *Ancylostoma* spp. from the environments actively penetrate human skin causing human cutaneous larva migrans [43, 44]. Furthermore, humans can be accidental hosts for *D. caninum* if they ingest infected fleas during the cysticercoid stage of pathogen development [37, 45]. In general, some human parasitic infections occur asymptomatically in humans, but others can cause serious illness and even death [37, 46, 47].

Efforts to prevent human disease include the appropriate treatment of pets [12, 28] and public education about the zoonoses of parasites [16]. In particular, the Veterinary Teaching Hospital in Indonesia plays a key role in preventing zoonotic parasite transmission, as they treat animals and provide public education, particularly for pet owners. To educate owners and veterinary professionals about helminth parasite prevention, up-to-date knowledge on gastrointestinal helminth parasites in pets is crucial.

This investigation conducted a retrospective analysis of the laboratory examinations for helminth infections in cat and dog patients with a parasite infection at the Veterinary Teaching Hospital of IPB University, Bogor, Indonesia.

## Materials and Methods

### Ethical approval

The research procedures have been approved by the animal care and use committee of the Veterinary Teaching Hospital of IPB University with ethical approval number 20–2016. The manuscript contained a study based on secondary data; therefore, no animal was used as an experimental animal in the trial. The fecal samples as the object for examination were collected by well-trained professionals (veterinarians or veterinary paramedics) concerning animal welfare regulations.

### Study period and location

The study was conducted to collect and analyze the available data of Helminthology Laboratory test results from January 2014 to April 2019. The data were obtained from patients’ medical records at the Veterinary Teaching Hospital of IPB University, Bogor, Indonesia.

### Data collection

A retrospective study design was used in this investigation. Helminth infections of dogs and cats were obtained from the patients’ medical records.

The Veterinary Teaching Hospital of IPB University receives patients to assess suspected helminth parasite infections (patients that have signs of cachexia, abnormal consistencies and color of feces, watery or bloody diarrhea, abdominal pain, and vomit worms), and for general check-ups, which include fecal examinations for parasites. Fecal samples were collected from the rectum using a spatula. A direct fecal examination was used to detect parasitic infection. This study used a wet mount fecal test to detect parasitic eggs. A small amount of the feces was placed on a microscope slide, a drop of aquadest was added, and it was then covered with a cover glass. The entire fecal smear was systematically examined for helminth parasite ova under a light microscope (Olympus BX51, Olympus Corporation, Japan) with 100×, 200×, and 400× magnification.

### Statistical analysis

The data obtained were analyzed using descriptive analysis to determine the prevalence of each helminthiasis in the dog and cat samples. The pet species, age, sex, breed, and helminth parasite infection status data were then analyzed using the Chi-square test to identify the risk variables for helminth parasite infections in pets.

## Results

In total, 54 dogs and 171 cats were examined for intestinal parasites. Of these, 61.11% of dogs and 53.80% of cats were positive for gastrointestinal helminthic infections (Table-1). The gastrointestinal helminths recorded in the dogs and cats in this study were ascarid (*Toxocara* spp.), hookworms, and *D. caninum*. Dogs were positive for *Toxocara* spp. (24.07%) and hookworms (37.04%) (Table-1), while cats were positive for *Toxocara* spp. (38.01%), hookworms (11.11%), and *D. caninum* (4.68%) (Table-1).

**Table-1:** Prevalence (%) of parasitic eggs in dog and cat faeces in the Veterinary Teaching Hospital of the School of Veterinary Medicine and Biomedical Sciences, IPB University, Bogor, Indonesia.

Animal species	Number of examined animals	Hookworm positive (%)	*Toxocara* spp. positive (%)	*Dipylidium caninum* positive (%)	All helminths positive (%)
Dog	54	20 (37.04)	13 (24.07)	0 (0.00)	33 (61.11)
Cat	171	19 (11.11)	65 (38.01)	8 (4.68)	92 (53.80)
Total	225	39 (17.33)	78 (34.67)	8 (3.56)	125 (55.56)

Age was found to influence the prevalence of hookworms in the dogs and cats (Tables-2 and 3). Older dogs and cats (>1 year) had a more significant risk of hookworm infection than younger ones (p < 0.05). Ascarids were detected more frequently in dogs and cats that were <1 year of age (p < 0.05). Furthermore, ascarid infections were also found to be influenced by breed (Table-2), while the prevalence of *D. caninum* was associated with cat age (p < 0.05) (Table-3). Sex did not significantly affect helminth parasite prevalence in the dogs or cats (Tables-2 and 3).

**Table-2:** Prevalence of helminthic infections in pet dogs based on different risk factors.

Risk factors	Categories	Number of examined animals	Number of infected animals	Prevalence (%)	Chi-square	p-value
Hookworms						
Sex	Male	25	11	44.0	0.968	0.325
	Female	29	9	31.0		
Age	Young (≤1 year)	25	4	16.0	8.835	0.003*
	Adults (>1 year)	29	16	55.2		
Breed	Pure breed	35	15	42.9	2.576	0.276
	Local	8	1	12.5		
	Mix breed	11	4	36.4		
*Toxocara* spp.						
Sex	Male	25	5	20.0	0.423	0.516
	Female	29	8	27.6		
Age	Young (≤1 year)	25	13	52.0	19.861	0.000*
	Adults (>1 year)	29	0	0.0		
Breed	Pure breed	35	4	11.4	10.433	0.005*
	Local	8	5	62.5		
	Mix breed	11	4	36.4		

* Significant at p *<* 0.05

**Table-3:** Prevalence of helminthic infections in pet cats based on different risk factors.

Risk factors	Categories	Number of examined animals	Number of infected animals	Prevalence (%)	Chi-square	p-value
Hookworms						
Sex	Male	81	8	9.9	0.238	0.626
	Female	90	11	12.2		
Age	Young (≤1 year)	120	7	5.8	11.348	0.001*
	Adults (>1 year)	51	12	23.5		
Breed	Pure breed	65	9	13.8	0.795	0.672
	Local	64	6	9.4		
	Mix breed	42	4	9.5		
*Toxocara* spp.						
Sex	Male	81	30	37.0	0.062	0.803
	Female	90	35	38.9		
Age	Young (≤1 year)	120	59	49.2	21.248	0.000*
	Adults (>1 year)	51	6	11.8		
Breed	Pure breed	65	20	30.8	2.339	0.311
	Local	64	27	42.2		
	Mix breed	42	18	42.9		
*Dipylidium* caninum						
Sex	Male	81	2	2.5	1.684	0.194
	Female	90	6	6.7		
Age	Young (≤1 year)	120	0	0.0	19.747	0.000*
	Adults (>1 year)	51	8	15.7		
Breed	Pure breed	65	6	9.2	4.911	0.086
	Local	64	1	1.6		
	Mix breed	42	1	2.4		

* Significant at p *<* 0.05

## Discussion

This study examined helminth cases recorded from January 2014 to April 2019 at the Veterinary Teaching Hospital of the School of Veterinary Medicine and Biomedical Sciences, IPB University. The overall prevalence of helminthic infections in dogs and cats was 61.11% and 53.80%, respectively. The prevalence was high compared to other parts of Indonesia (15.09%–57.14%) [48–50] or other countries, such as Ghana (52.6%) [51] and Thailand (40.1%) [52]. Lower prevalence levels have also been reported in cats in other studies from Indonesia (1.7%–37.8%) [53–55] and Thailand (33.9%) [52]. The prevalence results for dogs and cats in this study were lower than the results recorded for Klang Valley, Malaysia, which were 87.7% and 57.9%, respectively [56]. The differences in prevalence may be because the previous studies focused on stray dogs and cats, which have an increased risk for helminth infection compared to pets. In general, helminthiasis in dogs and cats may be related to management issues, such as being reared to be free-roaming, low hygiene and environmental conditions in their house, irregular deworming, lack of vector control, and low awareness in carrying animals [57].

The most frequently encountered parasites in this study in dogs and cats were hookworms and ascarids (*Toxocara* spp.). These results were in agreement with those for cats in Surabaya [53], Belgrade (Serbia) [58] and dogs and cats from metropolitan and micropolitan areas in Northern Italy and the Selangor and Pahang states of Malaysia [59, 60]. The distribution of the two types of parasites is a cosmopolitan [33, 34, 53, 56, 61–64], and generally, such parasites have a simple life cycle, direct transmission through contact or ingestion of infective larvae or eggs, and the presence of vectors [11, 61, 65, 66].

In this study, hookworm cases in cats (11.11%) were lower than those in dogs (37.04%). This result is similar to a previous study in cats and dogs (15.26% and 20.23%, respectively) in Guangdong, China [8]. Dogs may have higher infection rates than cats due to their rearing system and behavior. Dogs are generally tied up, kept outside the house, and more active than cats. Cat hookworm cases in this study were lower than those previously reported in Bali (37.59%) [67] but higher than those in Banyuwangi (3.96%) [68]. The prevalence of hookworms in dogs in this study is consistent with reported in Bali (34%) [69] and Cebu, in the Philippines (38%) [70]. A high prevalence of hookworm infection was previously found in dogs originating from Java Island (88.64%–92.5%) [71]. However, as the dogs in the previous study were local pups that were raised as roaming dogs, this may have affected the data. For example, the feeding behavior of stray dogs outside of the house increases their risk for hookworm infection [8]. Hookworm distribution worldwide among dogs and cats ranges from 15.26% to 95.8% [8, 52, 56]. In addition, another study by De *et al*. [72] reported a hookworm prevalence of 13.20% in dogs in Ludhiana, India. Different geographic areas and seasonal conditions may thus influence the variability in the prevalence of hookworm infections. For instance, India has four seasons, and the highest levels of hookworm prevalence occur in the autumn period, with a temperature range of 23°C–30°C; this creates favorable conditions for the pre-parasitic stage of development.

In this investigation, the prevalence of toxocariasis in cats (38.01%) was found to be lower when compared with the previous reports in other regions of Indonesia (43.65%–71.43%) [67, 68], but higher than other Indonesian regions [73–75]. The differences in prevalence may be related to rearing management, as high prevalence levels for toxocariasis were observed in Banyuwangi and Bali in stray cats. Globally, the prevalence of *Toxocara* worldwide is highest in strays compared to pets or working dogs, such as hunting dogs [76, 77]. *Toxocara* eggs can contaminate an environment and survive for long periods in feces, which increases the communication risk for other susceptible animals [61, 68]. Contamination with infective *Toxocara* eggs can also occur in the hairs of cats and dogs [40, 78–80] and children’s playgrounds worldwide [81–83]. This may be related to parasite biology, as female *Toxocara* worms can produce 200,000 eggs daily. These eggs become infectious within 1 month after being expelled in the feces [84]. Furthermore, particularly in dogs, there is the possibility of prevalence overestimation for toxocariasis due to their coprophagy behavior (consumption of their own feces). Dogs can thus act as transport hosts for all helminth eggs; this behavior can influence the results of the helminthic egg detection tests [85, 86].

This study reports that the prevalence rate *D. caninum*, which is 4.68% for cats, is lower than the reported rate for stray cats in Semarang (6.6%) [87] but higher than the reported rate in Vladivostok, Russia (2.2%) [88]. Further, the *D. caninum* infection rate is higher in older cats than in younger ones. Cats can be infected when ingesting fleas (*Ctenocephalides felis*) containing *D. caninum* larvae [89]. The risk of *D. caninum* infection increases in animals parasitized with lice or fleas [37, 90] as the intermediate hosts of *D. caninum* are dog and cat fleas (*Ctenocephalides* *canis* and *C. felis*, respectively) and cat and dog chewing lice (*Felicola subrostratus* and *Trichodectes canis*, respectively), while the final hosts are the dogs and cats. Infection in humans occurs when they accidentally ingest fleas infected with *D. caninum* cysticercoid [35, 91]. The low prevalence of *D. caninum* in cats in this study can be attributed to modern housing arrangements and enhanced environmental hygiene awareness in most homes, making it difficult for intermediate hosts to survive. Thus, the transmission cycle breaks the confined endemic focus of infection. In addition to using anthelmintics in infected cats and dogs, an important control method of *D. caninum* infection in cats and dogs involves the elimination of ectoparasites that are intermediate hosts [92–94].

Principally, hookworms can infect animals of all ages [95]. However, this study, has reported a higher prevalence of hookworm in older animals than in younger ones. Similarly, another study by Johnson *et al*. [96] reported a higher prevalence rate of hookworm infection (90.4%) in adult dogs (>12 months old) in Ghana when compared with young adult dogs (6–12 months old) (72.8%) and puppies (<6 months old) (40.7%). However, other studies have also reported contrasting results [7, 97, 98]. The differences in prevalence between different ages in different studies may depend on the parasitic intensity of infection and the locations of the sampled animals [99]. The differing results may also be related to the dog management strategy. The previous study has reported that most dogs are raised as free-roaming, hunting dogs raised outdoors. Hunting dog owners in Sukabumi usually go hunting with their dogs twice a week, and infection might occur during these events [100].

During the life cycle of *Ancylostoma caninum*, a hookworm infection can occur during the latent stage, larval hypobiosis, fecal–oral transmission, and mammary–oral infection [101]. Hookworm infection can occur when *A. caninum* infective larvae invade through the skin’s sweat glands, hair follicles, or through direct ingestion [70]. In addition, puppies can be infected through their mother through transplacental and trans mammary routes [78]. The larvae of *A. caninum*, as the causative agent of hookworm infection, can be reactivated during the animal host gestational period. These larvae can cause autoinfections of the dam and cause patent hookworm infections in adult animals [95], and in the offspring. Severe hookworm infection can cause anemia and death due to the resulting blood loss [10].

The prevalence of toxocariasis was higher in younger dogs and cats than in older ones. This is in line with the previous research [68], which has reported a higher disease prevalence in cats under 6 months, and a meta-analysis that showed a higher prevalence of toxocariasis in cats under 12 months when compared to cats aged >12 months [79]. This may be due to the transmammary transmission route in young animals [61]. Due to the transplacental infection of the fetus and the established evidence of age-associated immunity in adult dogs, as previously reported, it was hypothesized that the prevalence of *Toxocara* was more significant in young puppies. Older dogs may have established specific immunity to *Toxocara* due to frequent exposure at a young age [51]. Higher toxocariasis prevalence in young animals may be due to the immunity status, drug resistance, lack of anthelmintic therapy, and lack of owner awareness regarding parasitic infections [57].

In this study, there was a higher prevalence of *Toxocara* in dogs from local breeds; this may be because they are generally free-roaming, which frequently exposes them to contaminated environments [80]. *Toxocara* infections can be transmitted through the soil; and habits like feeding off floors and sleeping without a mat enable transmission through this route [51]. Studies have also reported the existence of non-embryonated and embryonated *Toxocara* eggs on dog hair [78, 80]. The community generally raises local dogs in low and middle economic incomes; consequently, their awareness of dog health conditions, including hygiene, nutrition, and deworming programs, is low [72].

The prevalence of gastrointestinal helminth parasites in cats and dogs does not show a significant difference between males and females. Differences in the susceptibility to parasitic worm infections based on sex remain controversial. A meta-analysis study reported a similar prevalence of toxocariasis in female and male cats [79]. Some studies have reported a higher prevalence of toxocariasis in female cats and dogs when compared with males [75, 98]. However, a meta-analysis of *Toxocara* infection in dogs showed contrasting results [76]. During pregnancy, female animals often experience stress and spend much more time with their offspring, which may exacerbate re-infestation. This condition may be related to the high infection rates in young pets that may cause reinfection in older female animals [98]. In contrast, another study reported a higher prevalence in male cats than in females [73]. The risk of male cats being infected with *Toxocara* is 1.5 times that of female cats [73], as male cats are more likely to leave the house than females, particularly during the mating season. This behavior means that male cats will have an increased risk of exposure to *Toxocara*.

The helminth parasites found in this study, namely *Toxocara* spp., hookworms, and *D. caninum* in cats; and *Toxocara* spp. and hookworms in dogs, are all zoonotic and may be a public health concern. Pet cats and dogs can have high carrier rates for ascarid and hookworms and they can thus contaminate the environments with infective eggs and larvae. The presence of infective helminthic eggs and larvae in the environment could be a source of infection for humans, specifically children [53]. In addition, these eggs can persist as pathogens in the environment for years [95]. Thus, administering anthelmintics to kittens and puppies at <1 month is crucial to reducing helminth contamination. This phenomenon occurs because female worms can become pregnant and lay eggs as early as 3 weeks after the kittens and puppies are born. This information is essential for practicing veterinarians on the front lines to help prevent the spread of parasites via pets. They can have direct contact with the potential risk factors for their pet patients and may communicate directly with the pet owners. Practicing veterinarians must be aware of these illnesses and their zoonotic potential, and treat helminth infections in pets and instruct owners on how to reduce the risk of zoonotic transmission.

## Conclusion

The results of this investigation have shown that helminth infections are widespread in dogs and cats from Bogor, Indonesia. The most common nematodes in both species were *Toxocara* spp., hookworms, and cestodes. *Dipylidium caninum* has been found to have zoonotic potential, and the discovery of zoonotic helminth parasites in local pets is a cause for concern for the inhabitants of Bogor. As a result, initiatives will be required to teach pet owners how to prevent these parasitic diseases, particularly in younger pet animals with significantly higher prevalence rates.

This investigation had some limitations. The first is that a traditional method for detecting parasitic infection (wet mount fecal smear) was used with a low accuracy level. In addition, The prevalence of infection in this study could be underestimated because the examination of the sample was carried out only once. Furthermore, not all pets that come to the hospital are examined for helminth parasite infection and thus, the prevalence may be underestimated. The samples were only examined for suspected parasite infections, which may not reflect the true prevalence in the Bogor area. Future investigations should thus improve on the study design and use improved parasitology diagnostics to determine the true parasitic infection rates in pet cats and dogs. To improve the research design, future studies should consider the detection of parasitic infections on all patients who come to the Veterinary Teaching Hospital, not only those with clinical signs or full medical check-ups, or epidemiological studies that can be designed to detect parasitic infections from samples representing the population of pet cats and dogs in Bogor. In addition, to improve the detection methods, future studies should also consider using more accurate diagnostic tools, such as flotation, sedimentation, or molecular techniques, to increase the accuracy of parasitic detection, as well as consider taking the fecal sample for 3 consecutive days from all patients to prevent prevalence overestimation due to the coprophagy behavior of dogs.

## Authors’ Contributions

YR: Study design, interpreted the data, and drafted the manuscript. TITD: Collected the data from medical records, interpreted the data, drafted, and revised the manuscript. ES: Analyzed and interpreted the data drafted, revised the manuscript. NGB: Collected the data from medical records and drafted and revised the manuscript. All authors have read, reviewed, and approved the final manuscript.
